# Artificial Intelligence Algorithms in Health Care: Is the Current Food and Drug Administration Regulation Sufficient?

**DOI:** 10.2196/42940

**Published:** 2023-01-16

**Authors:** Meghavi Mashar, Shreya Chawla, Fangyue Chen, Baker Lubwama, Kyle Patel, Mihir A Kelshiker, Patrik Bachtiger, Nicholas S Peters

**Affiliations:** 1 University College London NHS Foundation Trust London United Kingdom; 2 Faculty of Life Sciences and Medicine King’s College of London London United Kingdom; 3 School of Public Health Faculty of Medicine Imperial College London London United Kingdom; 4 School of Clinical Medicine University of Cambridge Cambridge United Kingdom; 5 Sloan School of Management Massachusetts Institute of Technology Cambridge, MA United States; 6 National Heart and Lung Institute Faculty of Medicine Imperial College London London United Kingdom

**Keywords:** artificial intelligence, machine learning, regulation

## Abstract

Given the growing use of machine learning (ML) technologies in health care, regulatory bodies face unique challenges in governing their clinical use. Under the regulatory framework of the Food and Drug Administration, approved ML algorithms are practically locked, preventing their adaptation in the ever-changing clinical environment, defeating the unique adaptive trait of ML technology in learning from real-world feedback. At the same time, regulations must enforce a strict level of patient safety to mitigate risk at a systemic level. Given that ML algorithms often support, or at times replace, the role of medical professionals, we have proposed a novel regulatory pathway analogous to the regulation of medical professionals, encompassing the life cycle of an algorithm from inception, development to clinical implementation, and continual clinical adaptation. We then discuss in-depth technical and nontechnical challenges to its implementation and offer potential solutions to unleash the full potential of ML technology in health care while ensuring quality, equity, and safety. References for this article were identified through searches of PubMed with the search terms “Artificial intelligence,” “Machine learning,” and “regulation” from June 25, 2017, until June 25, 2022. Articles were also identified through searches of the reference list of the articles. Only papers published in English were reviewed. The final reference list was generated based on originality and relevance to the broad scope of this paper.

## Introduction

Machine learning (ML) technology aims to improve the quality and efficiency of health care within the current health systems. Its applications encompass roles traditionally undertaken by health care professionals, such as clinical triage at emergency departments, mammography screening, and diagnosis undertaken by radiologists [1,2]. In many studies, ML algorithms have outperformed clinicians, for instance, in chest radiograph interpretation, skin cancer diagnosis, and directing optimal treatment strategies for sepsis in intensive care [3,4].

ML-based adaptive algorithms have the ability to *learn* and optimize their performance within the ever-changing clinical environment. The adaptability helps to optimize its clinical utility but has the potential to impact patient safety by introducing an element of unpredictability.

While there has been a significant increase in the volume of literature describing ML since 2010 [5], the regulation of adaptive ML technology has lagged behind its rapid technological advancement. In the United States, the current framework under the Food and Drug Administration (FDA) only regulates an algorithm at the point of clinical deployment but fails to account for the initial model inception, development, and evolution once deployed into clinical use. In the United Kingdom, the National Health Service (NHS) has accelerated its effort in digitalization within health care through the creation of NHSx and NHS AI Lab, with an emphasis on the development of a suitable governance framework for artificial intelligence (AI) in health care [6]. Elsewhere in the world, ML regulation is at varying stages. India does not draw a distinction between ML algorithms and other medical devices, while China’s New Generation Artificial Intelligence Development Plan does not address regulation of medical devices [7,8]. While the World Health Organization has published guiding principles for ML use, it does not outline a specific framework for regulation [9].

This paper aims to use the current FDA regulatory model as an example, build on the existing framework, and propose a novel regulatory pathway for ML algorithms from inception through clinical deployment to model evolution. Since ML algorithms aim to support or, in certain cases, replace the role of medical professionals, we likened the regulatory pathway to those of medical professionals. We then discuss the associated challenges to its implementation and potential solutions to overcome the challenges.

## Current Regulatory Pathways and Potential Issues

**Figure 1:**
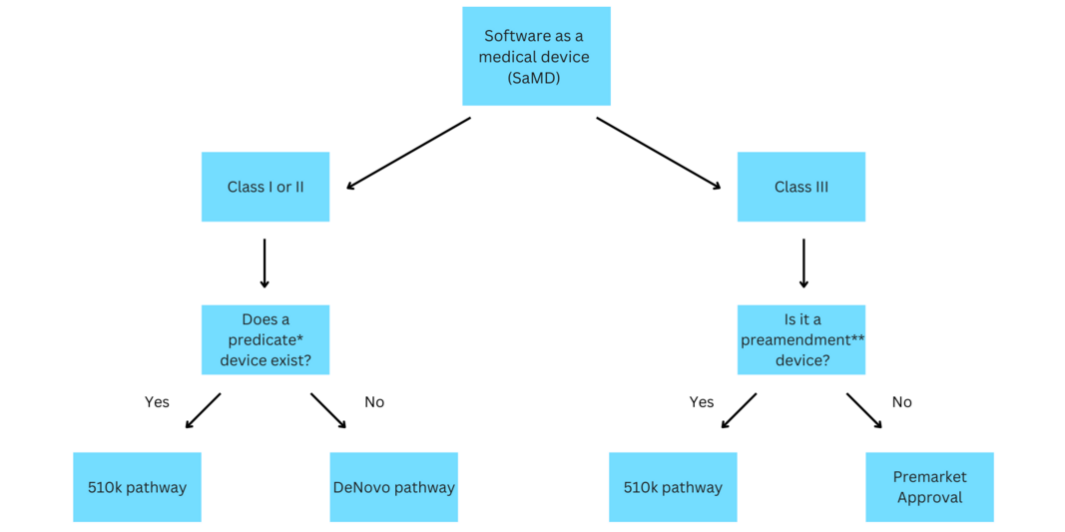
The current Food and Drug Administration (FDA) regulatory pathway. *A predicate: if the algorithm is found to be substantially equivalent to a legally marketed device. **A preamendment device: devices legally marketed in the United States before May 28, 1976, which have not been significantly changed/modified and for which no regulation requiring premarket approval has been published by the FDA.

Currently, most ML algorithms are approved by the FDA through one of three pathways: 510k, premarket approval, or the DeNovo pathway (see Figure 1) [10-12]. At a single timepoint prior to its approval, the ML production company will need to demonstrate the safety and effectiveness of the algorithm within its intended use. The current benchmark for approval requires companies to demonstrate good model performance on a varied data set and in a real-world setting. With no explicit definition of what constitutes reproducible standards, it is no surprise that the current FDA-approved ML algorithms vary considerably by the size of data sets and number of sites [5].

Under the current regulation, once an algorithm is approved, its behavior will remain fixed, defeating the distinguishing advantage of many adaptive algorithms in their ability to *learn* throughout their life cycle. Its current inflexible state not only reduces its clinical utility but can alarmingly infringe patient safety. For instance, an algorithm trained in 2018 to recognize pneumonia on a chest radiograph will not be able to differentiate it from COVID-19. Furthermore, variations exist in disease prevalence and population demographics across sites, such that the internal training and testing data sets used during algorithm development may not be representative of the population they are deployed to, thus performing poorly during external validation [13-15]. Moreover, depending on the training data set, the model may not be able to respond to geographically, ethnically, and socioeconomically diverse patient cohorts.

Additionally, the current FDA framework does not regulate the inception of an ML algorithm. As a result, a number of algorithms have been approved, many of similar use cases with varying development sites and data sizes. This can potentially constitute an inefficient use of resources [16].

## Current Attempts to Support Model Evolution

In April 2018, to account for the iterative improvement in ML model performance as new training data and improved data science techniques become available, the FDA released a white paper outlining a proposed framework for the regulation of ML-based software in medicine [17].

The proposed Total Product Lifecycle (TPLC) regulatory approach allows for iterative product improvement while maintaining essential safeguards. The framework adopts the principle of a *Predetermined Change Control Plan* produced by the manufacturer, which aims to anticipate potential modifications during clinical deployment. The Software as a Medical Device Pre-Specifications (SPS) will underline the modification expected by the manufacturer relating to *performance*, *inputs*, and *intended use*. Modifications within the SPS can be implemented without the need to resubmit for marketing application.

The implementation of the TPLC approach thus places the onus on the manufacturers to monitor and evaluate algorithm performance during its clinical use and regularly report to the FDA with updates and performance metrics. The culture of quality and organizational excellence of the company would be assessed according to the outlined standards in Good Machine-Learning Practice (GMLP). To date, only a single manufacturer of a cardiac ultrasound software has used the *Predetermined Change Control Plan* to facilitate future model alterations [18].

Elsewhere, similar trends have been observed in ML regulatory policies. The European Union has recently introduced the EU Medical Device Regulation, imposing stringent regulatory requirements from early-stage considerations through algorithm development to postmarket surveillance that need to be met prior to the clinical use of medical devices, including ML algorithms [19]. Likewise, in the United Kingdom, a code of conduct for AI and data-driven technology has been introduced to facilitate collaboration between technological companies and the NHS in developing high-quality safe medical devices [20].

While the TPLC approach has set out a useful theoretical framework in addressing the adaptive nature of ML algorithms, it places heavy emphasis on the manufacturer in governing the algorithm post deployment and overlooks the need to involve local end users immersed in the clinical environment. Moreover, despite being proposed for some time, the TPLC framework is yet to be implemented, which likely stems from the complexities involved. The framework also does not accommodate the evolution of algorithms beyond the predetermined specifications and change protocols. Finally, the framework has not addressed wider issues of the clinical utility, data suitability, and health equity of ML algorithms, which may call for a greater degree of regulation at a much earlier stage in the model life cycle.

In January 2021, the FDA released a document outlining an action plan in response to feedback from stakeholders on the TPLC approach, as well as SPS and the *Predetermined Change Control Plan* [21]. The five-point plan expressed the FDA’s intention to facilitate various enhancements to their TPLC approach, such as furthering GMLP by participating in communities (eg, the Xavier AI World Consortium) that collaborate to promote best practices in ML. In addition, the document expressed an appreciation for the need for a *patient-centered approach* as well as the evaluation of real-world model performance. The current action taken includes working with volunteers and engaging in further research to consider methods for real-world performance monitoring. Therefore, while the FDA has acknowledged many of the stakeholder queries (including some mentioned in this paper), there still is not much in the way of tangible solutions.

## The Proposed Regulatory Pathway

**Figure 2:**
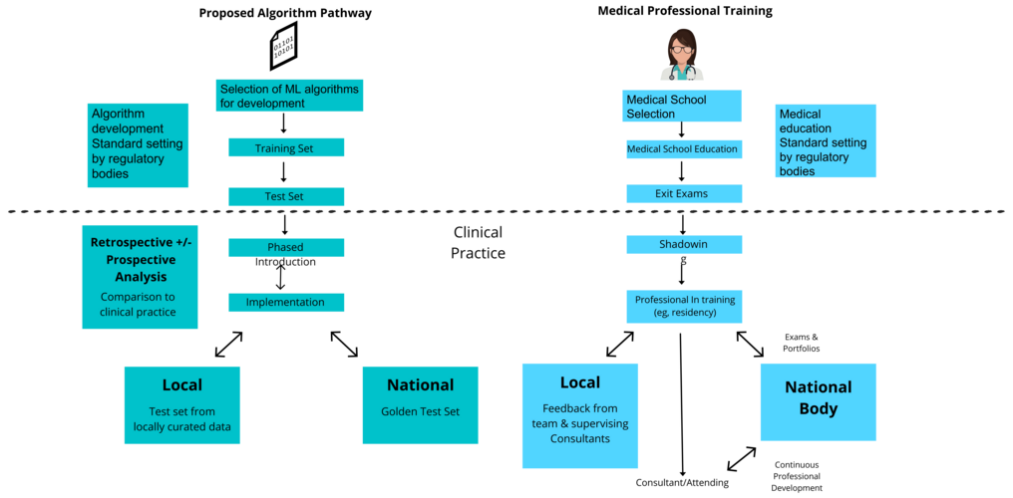
The proposed algorithm regulatory pathway analogous to the current medical professional training pathway. ML: machine learning.

Currently, the regulation of ML algorithms is akin to those for drug development [22]. However, the lack of ongoing prospective evaluation of AI algorithms truly limits their use in practice. As such, ML algorithms share a greater analogy to medical professionals, as they often undertake or support tasks traditionally performed by them and are subject to ongoing regulation. We therefore propose an analogous regulatory framework for ML algorithms, as summarized in Figure 2.

At the start, aspiring medical professionals are required to go through a selective process that ensures their baseline capabilities and suitability to begin their medical education. Similarly, the inception of an ML algorithm begins with a clinical problem that it aims to solve in health care. Algorithms across health care fields should be contested on their clinical value, usability, cost-effectiveness, and sustainability prior to its development, which will help to direct resources appropriately.

The model development phase can be likened to the undergraduate training of medical professionals. In the United Kingdom, the General Medical Council sets out standards and expected outcomes for medical education across the 44 recognized medical schools [23]. Similarly, in the face of the current heterogeneity present in the approved ML algorithms, structured standard-setting by an independent regulatory body should be in place during the development of algorithms on indicators such as data size and quality, technical assurance, and clinical safety. Guidelines such as TRIPOD-AI (Transparent Reporting of a Multivariable Prediction Model for Individual Prognosis or Diagnosis–Artificial Intelligence) and PROBAST-AI (Prediction Model Risk of Bias Assessment Tool–Artificial Intelligence) are being developed to help appraise AI-based prediction and diagnostic models [24]. These can be incorporated into the regulatory framework.

Prior to clinical deployment, the final clinical efficacy of ML algorithms is determined by a test data set, akin to the exit examination undertaken by medical professionals prior to qualification and employment. We propose further stages after the current regulation that ends after clinical deployment of ML algorithms.

Medical professionals often enter a period of supervision prior to independent practice, for instance, the internship period (foundation program) in the United Kingdom, which allows them to adapt to clinical practice [25]. Similarly, we propose that ML algorithms should enter a period of phased introduction that will involve an initial trial period for the algorithm to *observe*, operate alongside clinicians, and adjust to local working practices and systems. Ongoing evaluation and adaptation will take place in preparation for its full deployment.

After the initial period of shadowing, medical professionals are continuously re-evaluated to demonstrate ongoing competencies at a local and national level through national body board examinations [26], continuing professional development, and clinical portfolios [27,28]. We propose analogous local and national regulations for ML algorithms to ensure that they are consistently pertinent and useful in the ever-changing landscape of clinical practice. Locally, we propose for institutions to curate their own test sets containing representative cases that better reflect the variability in equipment, protocol, epidemiology, and patient populations encountered at the deployment site. Should the local testing demonstrate deficiencies, models can then be retrained on the curated local training data sets.

During the local training process, it is imperative that the algorithm does not deviate substantially from its initial objectives and continues to provide its proposed clinical benefit. While the FDA has delegated the task of ongoing data collection and monitoring of the algorithms to the manufacturer, a dedicated national regulatory body may be more suitable for this role. We thus propose the formation of national governance structures consisting of a panel of appointed experts who would be responsible for the selection of a series of cases that would typify the minimum standard the algorithm is expected to achieve within its specified case use—known as the *golden* test set. Unlike the local regulatory bodies, the core aim is to maintain safety and basic competence of the algorithm rather than its optimization. Additionally, the national *golden* test set will be updated in response to large changes in clinical practice by selecting cases from local representative data sets, for instance, when more effective treatment emerges, such as the use of mechanical thrombectomy for patients with stroke [29]; changes in policies, such as radiology imaging guidelines; and changes in pathology, such as the COVID-19 emergency.

## The Complexity Behind ML Regulation: Now and the Future

Both technical and nontechnical barriers pose a challenge to the implementation of any effective regulatory model. This may also explain why the TPLC approach has not been implemented more than 3 years following its inception. Ongoing regulation of an ML algorithm requires mechanisms to monitor model performance and methods of updating the model, the latter necessitating data sharing.

### Facilitating Model Evolution

Model drift, a process where the model’s prediction power deteriorates due to changes in the clinical environment, is the main cause of deviation in model performance once deployed clinically. The proposed regulatory pathway aims to engineer a performance monitoring and adaptation system on a local and national level that aims to detect, monitor, and mitigate the effect of model drift. Logistically, this process can be more nuanced.

In some circumstances, model drift can be anticipated, enabling retraining in advance of its occurrence. This is typically limited to foreseeable changes that alter the data distribution, such as a newly acquired computed tomography (CT) scanner that enables thinner reconstruction of images (eg, 1-mm thickness slices rather than 5-mm thickness). When the data in the domain is expected to change frequently, the identification of model drift can be automated so that the model can be retrained accordingly on a regular basis, both of which will require overhead infrastructure to be in place [30].

In other cases, once model drift is detected, its cause must be understood to take appropriate action. These range from biological factors such as a change in the characteristics of the patient population or management guidelines, technological factors such as novel treatment and imaging technology, and operational factors such as a change in the format of incoming data (eg, when the oxygen saturation probe outputs saturation as an integer [“97”] rather than a string [“97%”]).

To retrain ML algorithms, a wide range of methods are available from simple calibration to full retraining with the possible addition of new features. The choice of using old or new data for retraining depends on the application of the algorithm. For instance, if a specific cause has resulted in model drift, such as the above example of a novel CT scanner, then the model will need to be retrained on the new data as they are generated. If the drift is infrequent, both data sets can be combined to update the existing algorithm or generate a new algorithm. If the data is highly dynamic, retraining can be performed on new data while replacing the old.

During the retraining process, one must strive for a fine balance between maintaining the algorithm’s original function while adapting to its new local environment and minimizing new bias. For instance, in approaches that disregard the old data or algorithm, a risk of overfitting is present such that the algorithm may lose its original function. At the same time, care must be taken not to bias the model toward the outliers in the data set. For instance, the addition of a new data set with more cases of malignant chest nodules may bias an algorithm to predict lung cancer rather than benign modules from chest radiographs.

### Ground-Truthing

During the local and national testing and retraining, ground-truthing, or annotation of data to compare with algorithm predictions, is an essential process during model evolution. While fully automated methods exist, typically achievable in binary classification tasks with well-structured data, more complex tasks such as segmentation tasks (eg, identification of a lesion on a scan) will require manual labeling by a specialist. The question remains as to who, when, and how this process will take place alongside the clinical workflow. Independent companies that specialize in data annotation and ground-truthing exist, which may help to circumvent this added layer of complexity.

### Data Sharing

Insufficient sample size or restricted data sets can make it difficult for data to be interpreted through ML techniques subsequently introducing bias and underestimation of minority groups [31]. For example, the International Skin Imaging Collaboration: Melanoma Project, one of the largest dermatology data sets of pigmented lesions, largely focuses on Caucasian populations, which will limit its performance in other populations. Moreover, health outcomes are known to be worse in minority populations. Thus, it continues to be imperative to be able to acquire a range of data from a variety of sources to train ML models [32].

However, data sharing poses a challenge due to the sensitive nature of patient data and the sheer volume of data to be transferred [33]. During the current workflow for the development of ML algorithms, clinical sites typically share medical data for a specified period of time through two pathways: direct sharing and data enclaves. The former involves sending data out of the clinical network to the developers, while the latter takes the opposite approach by allowing external model developers into the clinical sites. Both routes can open up the potential for data misuse outside the agreed terms and compromise patient trust and safety. Data curated across multiple sites help to improve algorithm performance and minimize bias but will require greater stringency in its governance and standardization.

One potential solution is federated learning, a process that allows a model to be trained on multiple data sets across different sites by solely allowing access to specific features of each data set without physically exchanging data [34]. This circumvents the risks of data sharing while increasing the size and diversity of case pathology and demographics the algorithm is exposed to. Moreover, federated learning opens up the possibility of continuous learning by ongoing access to live data, rather than the outdated static data sets procured through the current two pathways. Collectively through a federated platform, the performance of the algorithm can be constantly tracked, trained, and tested.

Nevertheless, to unlock the full potential of this technique, we will need to overcome several logistical challenges. First, the initial algorithm development will still require intimate access to data. Second, data across sites can be stored in variable formats, making it more difficult to standardize and access the specified features required for federated learning. Finally, federated learning will need to be supported by adequate local hardware and networks, and can be bottlenecked by resource-constrained sites [34].

A number of ML- and non-ML–based prediction tools have been developed using national and international collaborative data sets [35-37]. In Taiwan, the National Health Insurance Research Database exemplifies a population-level data source for research in health care, with strict requirement for privacy and data confidentiality [38]. The Chronic Kidney Disease Prognosis Consortium, international collaborative data sets sponsored by the US National Kidney Foundation, harnesses data from over 80 population cohorts in an effort to improve the global outcome of kidney disease [39]. The use of data often requires stringent application through research institutions and public bodies. This, however, helps to optimize data quality, size, and diversity in a collaborative effort to direct ML technology toward priority areas while ensuring an optimal level of data governance.

### Integration Into Clinical Practice

Ultimately, the approved algorithms will need to yield sufficient clinical value to be accepted and integrated into the existing clinical workflow. Medical professionals will need to adapt their clinical practice and maximize the utility of the new technology. At the same time, ML algorithms make mistakes, as exemplified by the erroneous treatment recommendations made by IBM Watson for Oncology and the more recent Epic Sepsis Model that was found to miss two-thirds of sepsis cases that it was designed to predict [40,41]. Astringent safeguarding processes should be put in place, as the risk of faulty algorithms can affect a population at a system level, rather than of a single doctor-patient interaction [3].

#### Adaptation of Medical Professionals

The introduction of ML algorithms into the clinical workflow of medical professionals will not be an easy task. As mentioned above, we propose for a period of shadow deployment of the ML algorithm to allow clinicians to acclimatize to the new practice and troubleshoot for any issues while ensuring the algorithm is safe and reliable. During its clinical practice, once an algorithm is retrained, its functions and iterations may differ, while clinicians may continue to practice based on the algorithm’s prior behavior, introducing an element of automation bias. Therefore, clinicians will be required to continually adapt their clinical practice alongside the ML algorithm to maintain a good standard of care. Nevertheless, ongoing learning is already an integral part of medical professionals’ career paths. Clinicians have in the past adapted well to system changes such as the introduction of electronic health systems, the emergence of new diseases (COVID-19 being a stark example), alongside the flexibility in working with different members of the multidisciplinary team.

Looking beyond the future, the traditional health care training curriculum will need to adapt to the evolving medical technology through the introduction of ML into the medical curriculum. In fact, universities worldwide have recognized the demand for interdisciplinary medical professionals by introducing combined medical and engineering programs [42-44]. As proposed by Panch et al [45], ML may emerge as a new medical specialty to oversee the development and clinical implementation of ML algorithms into health care.

#### Adaptation of the Current Workflow

Ongoing local monitoring is a necessity. This will require design of a protocol and the use of specific resources. For instance, a threshold will need to be predetermined to trigger the re-evaluation of algorithm performance at a fixed interval or when a deterioration in performance is detected. When an algorithm is suspended for retraining and evaluation, a sustainable substitute will need to be in place to maintain the standards of care prior to its reintroduction.

The development of local test sets will become an additional process alongside the usual clinical practice. As to who will undergo the process of ground-truthing, the practice of internal clinicians that regularly work with the model may be influenced by the model itself, thus introducing bias to subsequent inputs. For instance, radiologists who rely on ML algorithms to detect nodules may be less adept at their detection during the ground-truthing process. On the other hand, external clinicians may be less accustomed to the local equipment and practices. The optimal solution may involve the recruitment of a representative number of internal and external clinicians to expose the algorithm to a variation in clinical practice and minimize bias. Nevertheless, the entire process of model evolution will require a learning curve for all health care workers involved.

#### Adaptation of the Governing Structure

At present, the FDA places the onus on the third-party manufacturers to develop, monitor, and evaluate their ML algorithms. This is no longer sufficient or efficient. As described above, independent local and national governing structures involving multiple stakeholders will need to be in place, taking on a strong oversight in regulating the development of algorithms, clinical implementation, detection of deviation in algorithm performance, curation of local and national data sets, and circumventing automation bias, all within the constraints of limited clinical resources. The governing responsibility should be shared among clinicians, managers, software engineers, parent company representatives, and patients.

#### Adaptation of the Health System

All local sites are not created equal. Smaller resource-limited hospitals with limited infrastructure or expertise may in fact benefit the most if the full potential of ML technology is used appropriately, supporting limited workforce resources, inefficient workflow, and inadequate time between patients and clinicians. These hospitals, however, will require extensive support. In addition, the potential increase in workload to facilitate the local evolution and monitoring of algorithms may be particularly taxing for smaller peripheral hospitals, potentially nullifying the local uptake of ML technology. Potential solutions may be in the form of a network of external ML experts as well as specialist hardware and software to support local implementation of ML algorithms, their monitoring, and evaluation. In addition, regulatory frameworks worldwide should emphasize the importance of equity and accessibility in the development of ML algorithms, taking into consideration resource-limited hospitals and countries, optimizing the use of available resources while optimizing the performance of the ML algorithms.

## Conclusion

The growing use and development of ML algorithms worldwide mandate the need for robust regulatory mechanisms. Current pathways proposed by the FDA demonstrate limited scope for the algorithm to adapt to the ever-changing clinical landscape. While propositions have been made on how to improve the existing pathways, they do not involve major stakeholders and face many challenges to implementation. Given the supporting role of ML algorithms alongside medical professionals, this paper has proposed a parallel regulatory pathway from inception to implementation that allows continuous model evolution throughout its clinical course. Complexities and barriers do exist in its implementation. Successful implementation will necessitate novel, robust, and ML-specific infrastructure and governing bodies. Concomitantly, adaptability of medical professionals and interdisciplinary collaboration will be vital to unleash the full potential of ML technology in health care while ensuring quality, equity, and safety.

## Abbreviations

AI: artificial intelligence
CT: computed tomography
FDA: Food and Drug Administration
GMLP: Good Machine-Learning Practice
ML: machine learning
NHS: National Health Service
PROBAST-AI: Prediction Model Risk of Bias Assessment Tool–Artificial Intelligence
SPS: Software as a Medical Device Pre-Specifications
TPLC: Total Product Lifecycle
TRIPOD-AI: Transparent Reporting of a Multivariable Prediction Model for Individual
